# Releasing interim results from a randomised clinical trial: an example from the QUARTZ trial

**DOI:** 10.1186/1745-6215-12-S1-A125

**Published:** 2011-12-13

**Authors:** Matthew Nankivell, Richard Stephens, Cheryl Pugh, Paula Mulvenna, Rachael Barton, Ruth Langley, Mahesh Parmar

**Affiliations:** 1 MRC Clinical Trials Unit, London, UK; 2 Freeman Hospital, Newcastle, UK; 3 Castle Hill Hospital, Hull, UK

## Introduction

QUARTZ is the first phase III randomised clinical trial of whole brain radiotherapy (WBRT) for patients with inoperable brain metastases from non-small cell lung cancer. It is designed as a non-inferiority trial to assess whether WBRT may be omitted without detriment to the patient’s survival while improving quality of life.

QUARTZ opened to recruitment in March 2007 and currently has 76 UK and Australian centres open. Despite universal support of the importance of the question and a number of major initiatives aimed at improving recruitment, by mid-2010 recruitment was slower than targeted and the trial was under threat of closure. It was suggested that one of the reasons for investigators not offering the trial to large numbers of their patients and for patients rejecting randomisation, was the lack of good quality preliminary randomised data to support the trial question. Therefore it was proposed to make the unusual step to release interim results from the trial in order to provide investigators with further information upon which to base trial decisions and discussions.

## Methods

The Trial Management Group and Trial Steering Committee made the decision to release the interim results. This decision was made without prior knowledge of the results. Data on the first 151 patients were analysed, and presented to investigators at a large meeting as part of a detailed presentation, with emphasis on the limitations of the data and guidance on interpretation. There are plans to publish a formal paper of the results (and the process) at a later date.

## Results

The response from attendees at the meeting was very positive. Attendees agreed that the results were not definitive in any way but provided a strong rationale for the trial continuing, to answer this longstanding clinical question. Recruitment has improved since the data release, from an average of 7.3 patients per month in July 2010, to an average of 10 patients per month by August 2011.

## Conclusions

There are dangers in releasing the interim results of a trial. However in this non-inferiority trial, it was judged that these dangers were outweighed by the dangers of the trial closing because of poor accrual. Release of the interim trial results in fact “saved” the trial from closure. We shall present more general principals of when release of interim trial results may be justified and useful.

